# The role of Eag and HERG channels in cell proliferation and apoptotic cell death in SK-OV-3 ovarian cancer cell line

**DOI:** 10.1186/1475-2867-11-6

**Published:** 2011-03-10

**Authors:** Viren Asher, Averil Warren, Robert Shaw, Heidi Sowter, Anish Bali, Raheela Khan

**Affiliations:** 1 School of Graduate Entry Medicine and Health Royal Derby Hospital, Uttoxeter Road, Derby DE22 3DT, UK; 2 Department of Gynaecological Oncology Royal Derby Hospital, Uttoxeter Road, Derby DE22 3NE, UK; 3 University of Derby Kedleston road, Derby, UK

## Abstract

**Background:**

The voltage gated potassium (K^+^) channels Eag and HERG have been implicated in the pathogenesis of various cancers, through association with cell cycle changes and programmed cell death. The role of these channels in the onset and progression of ovarian cancer is unknown. An understanding of mechanism by which Eag and HERG channels affect cell proliferation in ovarian cancer cells is required and therefore we investigated their role in cell proliferation and their effect on the cell cycle and apoptosis of ovarian cancer cells.

**Methods:**

The presence of Eag and HERG was determined in SK-OV-3 cells using immunofluorescence and western blotting. The effect of the Eag blockers (imipramine and clofilium) and HERG blockers (E-4031 and ergtoxin) on cell proliferation was assessed using the MTS assay with further investigation of their role in the cell cycle and apoptosis determined by flow cytometry.

**Results:**

Eag and HERG channels were present in the cytoplasm and nuclei of SK-OV-3 cells. There was significant inhibition of proliferation of SK-OV-3 cells by imipramine (P < 0.001) and ergtoxin (P < 0.05) at 72 hours of culture. Incubation of cells with ergtoxin led to the accumulation of cells in the S and G2/M phase, while cells accumulated in S phase after incubation with E-4031, with no effect on apoptosis. Imipramine did not affect the cell cycle but increased the proportion of SK-OV-3 cells undergoing early apoptosis.

**Conclusion:**

Both Eag and HERG channels are expressed in SK-OV-3 ovarian cancer cells and have a role in cell proliferation. HERG channels affect the cell cycle while Eag channels are implicated in the inhibition of apoptosis of ovarian cancer cells. The family of Eag channels may represent a new therapeutic target for the treatment of ovarian cancer.

## Introduction

Voltage gated potassium (K^+^) channels have been shown to be expressed in various cancers and appear to play an important role in progression of cells through the cell cycle, in part, due to the ion translocation across the plasma membrane [1].

The Ether-a-go-go (Eag) gene and Human Eag Related Gene (HERG) encode voltage gated K^+ ^channels belonging to the Ether-a-go-go family [2]. The Eag channel is aberrantly expressed in various cancers and has been shown to have oncogenic potential in heterologous systems [3]. Eag channels also affect the transition of cancer cells through the cell cycle [4]. Unlike Eag channels that are abundant in the central nervous system, HERG channels have been long known to play an important role in the repolarisation of the heart and are implicated in the molecular basis of familial Long QT2 syndrome [5]. HERG channels also play an important role in regulation of the cell cycle in various tumours [6]. Interestingly, a role for voltage gated K^+ ^channels in proliferation of ovarian cancer cells has been proposed following inhibition of proliferation with the non specific voltage gated blocker 4-aminopyridine (4-AP) that led to accumulation of cells in the G1 phase [7].

Ovarian cancer is the second most common female genital malignancy in the UK, accounting for 6% of female deaths due to cancer. Worldwide there are about 225,000 new cases of ovarian cancer of which 125,000 women die each year. The highest incidence of the disease is in Northern Europe and USA and the lowest in Africa and Asia [8]. Despite advances in chemotherapy, ovarian cancer mortality rates in the UK since the early 1970s, have remained stable at ~10- 12 per 100,000 women. This is in part due to the asymptomatic nature of the disease with most women presenting at a late stage [8]. Hence, there remains a need for the development of newer therapies based on new targets to improve survival. Given their association with various cancers, both Eag and HERG channels show promise as future therapeutic targets.

We have shown that Eag and HERG channels are expressed in SK-OV-3 ovarian cancer cells and their blockers significantly inhibit their proliferation. HERG blockers inhibit the proliferation at S and G2/M phase while Eag blocker impramine increases early apoptosis in ovarian cancer cells with no effect on cell cycle.

## Material and methods

### Cell line

The SK-OV-3 cell line (Passage 13) was kindly donated by Dr Dimitra Dafou, Translational Research Laboratories, University College Hospital, London in February 2009, from an authenticated stock provided by the American Type Culture Collection (ATCC). Cell cultures were grown in Roswell Park Memorial Institute (RPMI) 1640 medium supplemented with 10% fetal bovine serum (FBS) and antibiotics (100 u/ml penicillin and 100 μg/ml streptomycin; Invitrogen, Paisley UK).

### Antibodies and Drugs

Mouse anti-human Eag antibody was purchased from Abnova Labs (Taiwan). Rabbit anti-human HERG antibody was from Abcam laboratories (UK) while secondary anti-mouse and anti-rabbit fluorescein isothiocyanate (FITC) conjugated antibodies were obtained from Sigma (Poole UK). Imipramine and clofilium were obtained from Sigma Aldrich UK while E-4031 and ergtoxin were from Caltag Medsystems, UK.

### Immunofluorescence

The SK-OV-3 cells were grown on cover slips in 24 well plates and incubated for 24 hours until 80% confluent. Cells were then fixed with 4% paraformaldehyde for 20 min and treated with 0.5% Igepal before blocking with 3% BSA/5% glycine in phosphate buffered saline (PBS) followed by 10% goat serum in PBS. Eag and HERG (both 1:100 in 10% goat serum/PBS) antibodies were added to the wells for overnight incubation at 4°C. Wells containing 10% goat serum without the primary antibody were used as a negative control. After incubation, FITC conjugated secondary antibody (1:50) in 10% goat serum/PBS was added and cells incubated at room temperature for 90 min. Images of the cells were viewed on a fluorescence microscope (Axiovert, Zeiss) and captured using Cell F software (Olympus UK).

### Western blotting

The SK-OV-3 cells were initially washed three times with Hanks balanced salt solution (HBSS) before collection by scraping and lysis in homogenisation buffer [320 ηM sucrose, 10 mM Tris base (pH 7.8), 50 mM potassium chloride, 1 mM EDTA, 0.5% Igepal, 1:500 protease inhibitor cocktail and 1:100 phosphatase inhibitor cocktail II (Sigma Aldrich)] at room temperature. A crude cell lysate was obtained by centrifugation at 14,000 g for 60 min at 4 °C twice. Protein concentration was determined by the Bicinchoninic acid method with bovine serum albumin (BSA) as a standard. The protein samples (50 μg) were separated on 10% sodium dodecyl sulfate-polyacrylamide gel electrophoresis and then transferred to a nitrocellulose membrane. After being blocked in 10% milk protein (Marvel) in TBS-T (20 mM Tris, pH7.4, 500 mM NaCl, 0.01% Tween 20), the membrane was incubated overnight at 4 °C with Eag (1:100) and HERG antibodies (1:1000). The membrane was then washed six times with TBS-T for 10 min each and incubated with secondary antibody linked to alkaline phosphatase (Eag 1:1000 and HERG 1:1500, Sigma-Aldrich) for 90 minutes at room temperature. After washing six times with TBS-T again for 10 minute each, the blot was then developed using alkaline phosphatase substrate enhancer. Blots were viewed using the chemidoc imaging system (Bio Rad Labs).

### Cell proliferation assays

The effect of the Eag blockers (imipramine and clofilium) and HERG blockers (E-4031 and ergtoxin) on SK-OV-3 cells, was examined using MTS (3-(4, 5-dimethylthiazol-2-yl)-5-(3-carboxymethoxyphenyl)-2-(4-sulfophenyl)-2H-tetrazolium, inner salt) assay in the form of the CellTiter 96 aqueous non-radioactive cell proliferation assay kit (Promega, UK). The assay was performed according to the manufacturer's instructions. Briefly, SK-OV-3 cells were seeded at a density of 5000 cells/100 μl in RPMI 1640 medium containing 10% FBS in triplicate wells of a 96 well plate (Perkin Life Sciences) and incubated for 24 hours in 5% CO_2_/air at 37°C. Thereafter, the medium was aspirated and replaced with 100 μl fresh medium containing Eag and HERG blockers at various concentrations or medium only which served as a negative control. All drugs were reconstituted in RPMI 1640. Cells were incubated for 96 hours in the test drug and proliferation assessed daily by the addition of MTS reagent. After an hour's incubation, the plates were read at 490 nM using a Victor 1427 multilabel counter (Wallac). All assays were repeated thrice. The choice of drug concentrations used in the cell proliferation was based on previous published data for imipramine [9], clofilium [10], E-4031[11] and ergtoxin [12].

### Cell cycle assay

SK-OV-3 cells were seeded at a density of10^5^/ml of RPMI 1640 with 10% FBS in a 6 well plate and incubated for 24 hours in 5% CO_2_/air at 37°C. The medium was aspirated after 24 hours and replaced with fresh medium in the presence and absence of Eag blockers (imipramine 50 μM and clofilium 3300 nM) and HERG blockers (E-4031 20 μM and ergtoxin 100 nM). The plate was then incubated for 48 hours. 1 × 10^5^/ml cells were dispensed in FACS tubes, washed with PBS and fixed with 70% ethanol. 10 μl/ml of 10 mg/ml RNase solution (DNase free, Sigma-Aldrich?) was added and then cells stained with 50 μl/ml of propidium iodide (1 mg/ml, Sigma). Cells were incubated at 37°C for 20 min and fluorescence from 20,000 events determined using a Coulter Altra Flow cytometer. The population of cells in the G0/G1, S and G2/M phases were quantitated based on scatter analysis using WinMDI 2.9 and Cylchred software. The highest inhibitory concentrations of all drugs in cell proliferation experiments were used for cell cycle analysis and apoptosis assay.

### Apoptosis assay

Apoptosis was assessed using Annexin V binding with propidium iodide (PI) staining using the TACS Annexin V kit (Trevigen Inc). SK-OV-3 cells were initially seeded at concentration of 100,000 cells/ml, in 6 well plates and incubated at 37°C in a humidified atmosphere with 5% CO_2_/air. After 24 hours, the Eag blockers (imipramine 50 μM and clofilium 3300 nM) and HERG blockers (E-4031 20 μM and ergtoxin 100 nM) were added with one well containing medium only serving as a control. After exposure to the drugs for 48 hours, cells were dissociated using 0.05% trypsin/EDTA and then washed with cold PBS. approximately 100,000 cells were then suspended in 100 μl of Annexin V Incubation buffer containing 10 μl of 10 × binding buffer (100 mM Hepes, pH 7.4, 1.5 M NaCl, 50 mM KCl, 10 mM MgCl_2 _and 18 mM CaCl_2_), 10 μl of PI (50 μg/ml), 1 μl Annexin-FITC in deionised, distilled water. After incubation in the dark for 15 minutes at room temperature, binding buffer was added and cellular fluorescence analysed by the Coulter Altra Flow cytometer for 10,000 events. Control tubes containing binding buffer only and cells containing Annexin V alone and PI alone were initially used to calibrate the instrument.

### Statistical analysis

Statistical significance was determined using one way analysis of variance (ANOVA) with Dunnet's multiple comparison post hoc analysis (Graphpad Prism 5). All results are presented as mean ± standard error of mean (SEM), with data considered significant at P < 0.05.

## Results

### 1. Eag and HERG channels are expressed in ovarian cancer cells

**Figure 1:**
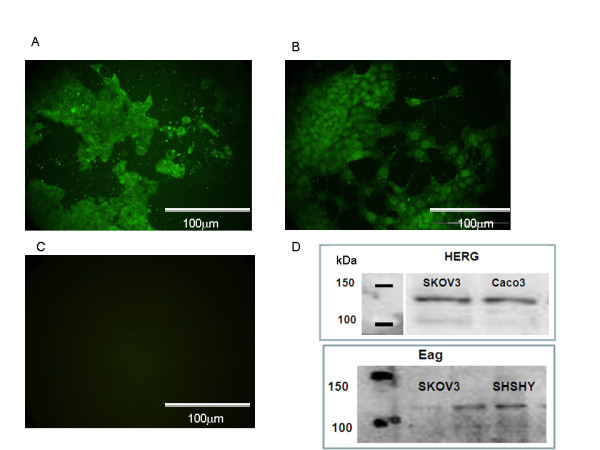
**Presence of Eag and HERG channels on SK-OV-3 cells**. Ovarian cancer cells demonstrate immunofluorescence staining predominantly in the nucleus and cytoplasm with both (A) Eag and (B) HERG antibodies (C) negative control where primary antibody is replaced with goat serum showing no immunofluorescence (D) Eag and HERG protein demonstrated by SK-OV-3 cells on western blotting. Neuroblastoma (SHSHY) and colon cancer (Caco3) cells lines were used as positive control for Eag and HERG respectively.

SK-OV-3 cells demonstrated positive immunofluorescence in the cytoplasm and the nucleus for both Eag and HERG channels as shown in Figure 1 (A and B). No immunoreactivity was observed in negative control experiments where primary antibody was replaced with goat serum, (Figure 1C). Eag and HERG protein expression in SK-OV-3 cells was alsodemonstrated by Western blotting (Figure 1D).

### 2. The proliferation of SK-OV-3 cells is significantly inhibited by both Eag and HERG blockers

**Figure 2:**
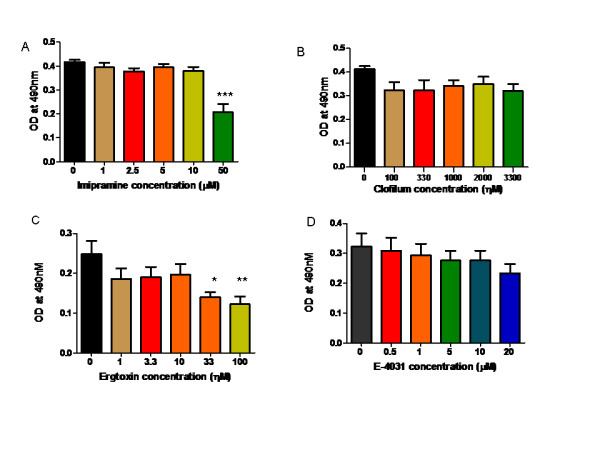
**Effect of Eag and HERG blockers on SK-OV-3 cell proliferation**. There was a significant inhibition of proliferation of SK-OV-3 cells on incubation with the Eag blocker imipramine at a concentration of 50 μM (A) and the HERG blocker ergtoxin (100ηM) (Figure 2C) (* P < 0.05, **P < 0.01, ***P < 0.001)

The Eag blocker imipramine at 50 μM significantly inhibited the proliferation of SK-OV-3 cell lines at 72 hours of culture (P < 0.001; Figure 2A) while clofilium (100-3300 nM) did not show any effect on proliferation at any of the time points tested ( Figure 2B). Ergtoxin significantly inhibited the proliferation of SK-OV-3 cells at 33 (P < 0.01) and 100 ηM P < .05) ( Figure 2C) while no significant effect of E-4031 on cell proliferation at any of the concentrations applied, was observed ( Figure 2D). All experiments were repeated thrice.

### 3. E-4031 and Ergtoxin inhibit proliferation through their action on the cell cycle

**Figure 3:**
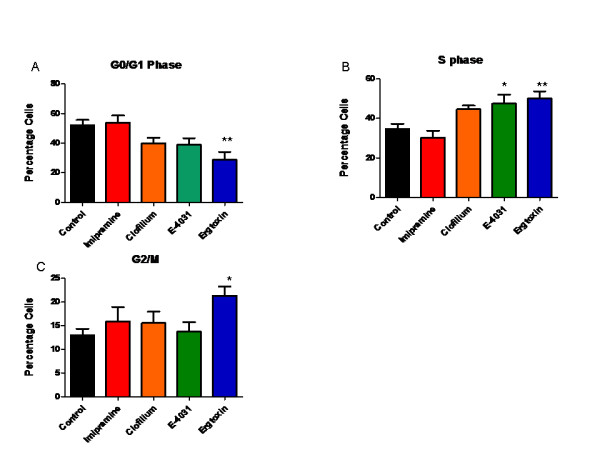
**Cell cycle analysis after incubation with Eag and HERG blockers**. Effect of Eag blockers (imipramine and clofilium) and HERG blockers (E-4031 and ergtoxin) on SK-OV-3 cells. E-4031 (20 μM) shows a block after S phase leading to increased cells in the S phase (Figure 3B) while ergtoxin (100ηM) has an effect on G2/M cell cycle checkpoint leading to the accumulation of cells in G2/M and S phase and less cells going through to G1 phase (Figure 3A-C) (* P < 0.05, ** P < 0.01). Imipramine (50 μM) and clofilium (3300ηM) showed no effect on cell cycle.

To elucidate the effect of these drugs on the cell cycle, we investigated the effect of Eag and HERG blockers on SK-OV-3 cells using flow cytometry. Both Eag blockers imipramine 50 μM and clofilum 3300 ηM demonstrated no effect on the G0/G1, S or G2M phases of the cell cycle (Figure 3A-C). E-4031 (20 μM) led to accumulation of cells in the S phase of the cell cycle (Figure 3B), while ergtoxin (100 ηM) a *Centruroides noxius *scorpion toxin, a known specific HERG channel blocker [13] increased accumulation of cells in S (Figure 3B) and G2/M phases (Figure 3C). The analysis of percentage of cells in various phases was done using Cylchred software

### 4. Imipramine affects the proliferation of SK-OV-3 cells through apoptosis

**Figure 4:**
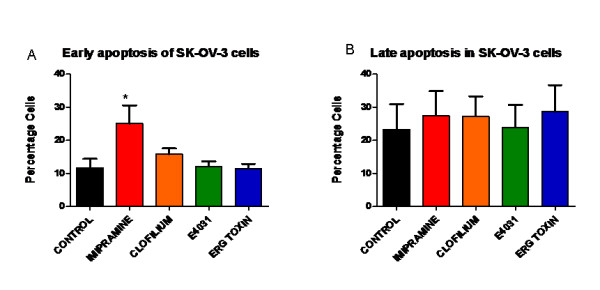
**Effect of Eag and HERG blockers on apoptosis of SK-OV-3 cell line**. Cells treated with imipramine (50 μM) showed significantly high number of cells programmed to undergo apoptosis compared to control cells (*P < 0.05), while clofilium (3300ηM), E-4031 (20 μM) and ergtoxin (100ηM) showed no effect on apoptosis.

Annexin V binding assay was used as it has been shown to be a good indicator for detection of cells that are programmed to undergo early apoptosis [14].Only cells treated with 50 μM imipramine underwent early apoptosis compared to control, as determined by Annexin V binding while 3300 ηM clofilium, 20 μM E-4031 and 100ηM ergtoxin had no effect on apoptosis (Figure 4A and 4B).

## Discussion

Eag and HERG channels are known to be associated with cell proliferation and cell cycle in various cancers [1,4]. Both of these channels belong to the same *ether-a-go-go *family and share a similar structure therefore any drug inhibiting Eag will potentially also affect HERG channels leading to undesirable cardiac side effects [15]. Imipramine has shown Eag blocking activity in HEK293 cells expressing Eag channels [9], while clofilium has been shown to block both Eag and HERG channels but is not considered HERG specific [10]. E-4031 has also been shown to inhibit HERG channels [16] while ergtoxin is a specific HERG blocker [13].

In our study, SK-OV-3 cells exhibited immunofluorescence for both anti-Eag and anti-HERG antibody. Immunostaining was localised to nuclei and cytoplasm providing support for the presence of these channels in ovarian cancer. Western blotting confirmed expression of both Eag and HERG in SK-OV3 cells. The exact function of Eag and HERG channels in the cytoplasm and nucleus is not known although its cytoplasmic expression may suggest retention due to impaired trafficking of this protein. Imipramine has been shown to inhibit various channels at IC_50 _values that range between 1- 30 μM [9], although we found that a higher concentration of imipramine (50 μM) was needed to demonstrate significant inhibition of proliferation of ovarian cancer cells compared with effects on whole-cell Eag currents [9]. Clofilium has been shown to block Eag channels expressed in *Xenopus *oocytes and HEK cells [10] but has not tested in cancer cells.

The lack of inhibition of proliferation of SK-OV-3 cells observed with E-4031, is in line with findings in breast cancer cells [11]. However, many other cancers that express HERG channels such as leukaemia [17] and colorectal cancer [18] have shown inhibition with E-4031. Ergtoxin due to its specificity of blockade of HERG channels significantly inhibited ovarian cancer cells. In contrast, ergtoxin did not affect breast cancer cell proliferation even at concentrations of 300 ηM [11]. Various mechanisms have been postulated to explain the effect of voltage gated K^+ ^channels on cell proliferation. The most accepted explanation suggested is through their effects on cell volume and membrane potential [19]).

In order to investigate if the anti proliferative effects of Eag and HERG channels were cell-cycle specific, we incubated the cells with the drugs in their highest inhibitory concentrations as tested in proliferation assays and carried out cell-cycle phase distribution analysis by flow cytometry. Neither imipramine nor clofilium had any demonstrable effects on the cell cycle although imipramine is associated with accumulation of cells in G0/1 phase in melanoma [20]. The effect of E-4031 showing accumulation of cells in S phase is in contrast to the effects of this drug in leukaemia and melanoma cells where it inhibited G1/S transition [17,20]

We have also shown that imipramine inhibits SK-OV-3 proliferation by inducing early apoptosis with no effect on cell cycle while clofilium, E-4031 and ergtoxin had no effect on apoptosis of SK-OV3 cells. Imipramine has been shown to induce apoptosis of melanoma cells at concentrations greater than 20 μM [20], but no effect on apoptosis was noted on neuroblastoma cells at 50 μM [21]. Clofilium has been shown to induce apoptosis in human promyelocytic leukaemia [22] but was used at a higher concentration (10 μM), than that used for our experiments. This may explain the lack of effect of clofilium on apoptosis in our system. E-4031 has shown no effect on apoptosis on leukaemic cells [17] which is consistent with our results.

Our results demonstrate that Eag and HERG blockers exert their antiproliferative effects through different mechanisms. The effects of imipramine on proliferation, though not cell-cycle specific, appeared to be through induction of early apoptosis via blockade of Eag channels while ergtoxin showed no effect on apoptosis but exhibited its antiproliferative actions by blocking cells in the G2/M phase. There are no specific blockers of Eag and HERG channels and both Eag and HERG blockers have cross reactivity with other K^+ ^channels [19]. The drugs used in our experiments have been shown to block specifically Eag and HERG channels in heterologous systems but there is a possibility that they may act via a different unidentified mechanism. Our inference from the experiments is the most plausible interpretation of findings given the available evidence. Although further detailed analysis of mechanisms underlying the effect of channel blockers in ovarian cancer is needed, the significance of targeting channels that determine key features such as cell size and membrane potential as they progress through the cell cycle offers new opportunities through which to manipulate proliferation and apoptosis. Indeed carboplatin, a chemotherapeutic agent is routinely used to treat ovarian cancer acts by inhibiting DNA synthesis and promoting apoptosis of cancer cells [23]. It is conceivable that drugs targeting the Eag channel family may potentially be combined with carboplatin and used in patients with recurrence to reduce the dosage of carboplatin and improve survival.

In conclusion, we have shown that Eag and HERG channels are present in SK-OV-3 ovarian cancer cells and have a role in their cell proliferation. Imipramine and ergtoxin affect proliferation by different pathways and have a potential therapeutic role in the treatment of patients with ovarian cancer.

## Competing interests

The authors declare that they have no competing interests.

## Authors' contributions

VA performed the experiments and wrote the manuscript, AW and HS helped in performing and planning the experiments, RWS, AB and RNK were involved in planning of the experiments and helped in correction of the manuscript. All authors have read and approved the final manuscript.
